# Conceptualising equity in basic medical education accreditation standards: A qualitative study of expert perspectives

**DOI:** 10.12669/pjms.42.4.14422

**Published:** 2026-04

**Authors:** Neelofar Shaheen, Usman Mahboob, Ahsan Sethi, Muhammad Irfan

**Affiliations:** 1 Neelofar Shaheen, MHPE. Institute of Health Professions Education & Research, Khyber Medical University, Pakistan. Department of Health Professions Education and Research, Peshawar Medical College, Riphah International University, Pakistan; 2 Usman Mahboob, MPH, FHEA, Doctorate HPE, Post-Doctorate, Fellow FAIMER. Institute of Health Professions Education, Khyber Medical University, Peshawar, Pakistan; 3 Ahsan Sethi, MPH, MMedEd, FHEA, MAcadMEd, FDTFEd, PhD, FAIMER Fellow Health Professions Education, Department of Public Health, College of Health Sciences, QU Health, Qatar University, Doha, Qatar; 4 Muhammad Irfan, MCPS, FCPS, Dip CBT, MS-Mental Health Policy & Services, PhD. Department of Mental Health, Psychiatry, & Behavioural Sciences, Peshawar Medical College, KPK - Pakistan. Riphah International University, Pakistan

**Keywords:** Accreditation, Basic Medical Education, Equity, Qualitative

## Abstract

**Objectives:**

Accreditation systems are crucial in shaping medical education programs globally; however, concerns exist regarding the equitable application of standards across diverse contexts. This study explored expert perspectives on the conceptualisation of equity in basic medical education accreditation standards.

**Methodology:**

This study employed a qualitative theory-informed analysis design, conducted from January 2024 to April 2025. One-to-one interviews were conducted with 14 experts from various geographical locations, all of whom were involved in accreditation as researchers, surveyors, or members of national or international accrediting bodies. The data were analysed using thematic analysis guided by Aristotle’s theory of equity.

**Results:**

Experts emphasised the need for context-responsive standards that maintain quality while allowing flexibility in implementation. Five key themes emerged: 1) equity or equality: what should be preferred; 2) quality over quantity and contextualisation; 3) equity is not lowering the standards 4) equity is flexibility in interpreting the standards and 5) striking balance between equity and equality: the way forward.

**Conclusions:**

By incorporating equity principles, accreditors can foster inclusive excellence across diverse medical education landscapes by embracing flexibility (equity to achieve equality), contextualisation, systematic surveyor training, and collaborative improvement without compromising the quality of medical education.

## INTRODUCTION

Accreditation systems are crucial in shaping medical education programs globally, significantly affecting the quality of medical education.^1^-^3^ Accreditation serves as quality assurance (QA) by serving as a link in the quality chain in which institutions, programs, or judged against predefined criteria.^4^ While accrediting authorities differ worldwide, medical schools also exhibit disparities in resource allocation, with some institutions facing disadvantages owing to their geographical location and other socioeconomic factors.^5^ The most recent 2020 revision of the WFME standards allows for local adaptation of standards based on the principles outlined.^6^

The concept of “contextualisation” in accreditation processes is gaining importance as a key factor in ensuring fair evaluation of medical schools across the globe.^7^,^8^ This approach considers the diverse contexts and resource availability of different institutions, moving away from a one-size-fits-all accreditation model.^9^,^10^ Factors such as geographical location, available resources, and local healthcare needs of the community significantly influence the functioning of medical schools,^11^ as these institutions cannot be viewed as isolated operational units. The term “equity” as conceived by Aristotle, is the justice that goes beyond the written law.^12^,^13^ Equity allows for the flexibility and consideration of unique circumstances when interpreting and applying laws, aiming to achieve a more just and fairer outcome.^13^ This idea recognises that laws cannot anticipate every possible situation, and sometimes, a broader understanding of justice is necessary to ensure truly fair treatment. Equity has been a component of law and justice for centuries, as demonstrated in Aristotle’s theory of equity,^14^ as it focuses on the principles of fairness, conscience, and good faith, allowing judges to consider the specific circumstances of each case.^13^ We propose that the concept of “equity” reinforces the concept of “contextualisation”, and can be considered a guiding principle in accrediting medical schools.

Local and regional accrediting agencies are granted autonomy to formulate their own standards, provided these standards are both comprehensive and suitable for the specific context in which medical education is delivered.^15^ The WFME explicitly encourages agencies that voluntarily adopt the BME template to adapt standards to align with local contexts.^6^ Research on practical application of accreditation principles would ensure that they are optimised for contextual implementation.^16^ While the existing literature has explored institutional accreditation documents and conducted comparative analyses,^3^ the perspectives of experts on the critical issue of adapting standards at both global and regional levels represent a unique area of enquiry.

Our study explores the perspectives of experts on the use of the term “equity” in BME accreditation standards. A critical consideration is whether schools, which are tasked with upholding the principles of equity in medical education,^17^ are themselves accredited based on principles of equity. Existing literature underscores the drawbacks of “one-size-fits-all” approach, which frequently places low-resource institutions at a disadvantage.^18^ This study, drawing on interactions with global accreditation experts, explored their views on adapting global standards and the challenges linked to local adaptation. The study’s objectives were to analyse experts’ perspectives on the description and conceptualisation of term “equity” in BME accreditation standards. We sought to address the following research question: How do experts in Health Professions Education (HPE) accreditation conceptualise equity, as distinct from equality, in BME accreditation standards?

## METHODOLOGY

The research team designed a qualitative study employing theory-informed analysis grounded in the constructivist paradigm. The study duration extended from January 2024 to April 2025. We recruited participants using purposeful sampling strategy. Twenty-four experts from various geographical regions were contacted via email, LinkedIn profiles, WhatsApp, and personal connections. Fourteen invitees consented to participate in the study. The sample was predominantly male (n=10, 71%) compared to female (n=4, 29%). All participants held senior academic positions. One-on-one interviews were conducted with accreditation experts from Pakistan (*n=2*), Canada (*n=2*), USA (*n=2*), and (*n=1*each) from Caribbean region, Iran, New Zealand, Saudi Arabia, Sri Lanka and UK. In addition to the concept of data saturation for determining sample size, we also adhered to concepts of information power,^19^,^20^ conceptual depth,^21^ and theoretical insufficiency^22^-^24^ to evaluate data collection and finalise the number of participants in the study.

Participation in the study required a PhD or active pursuit of doctoral studies in HPE, prior involvement with accreditation bodies as consultants, surveyors, or researchers, or facilitation of accreditation visits. Interview invitations, followed by reminders, were sent to the identified experts from Latin America, Africa, and South Asian countries. The experts were labelled as “did not respond” if authors did not receive reply after three weeks. The demographic details of the participants are presented in Table-I.

**Table-I:** The professional background, geographical location and experience of the participants.

S.No.	Academic designation	Professional & geographical background	Experience in accreditation
1	Professor	Healthcare quality management systems, Medical Education Egypt	Research in accreditation National accreditation project development
2	Professor	Neurophysiology Medical Education Saudi Arabia	Member of national accrediting agency Facilitator of accreditation visits Experienced as surveyor
3	Professor	Medical Education PhD Pakistan	Member of national accrediting agency Development of accreditation standards for Post graduate medical education, Development of survey instrument
4	Professor	PhD Medical Education USA	Member of international accrediting body, Research in accreditation
5	Professor	Medicine Medical Education Sri Lanka	Member of national accrediting unit Development of national accreditations standards
6	Professor	Medical Education United Kingdom	Standards development for postgraduate training and curriculum, experience with international accreditation organization
7	Professor	Medicine Medical Education Canada	Research in accreditation Undergraduate and postgraduate accreditation, Surveyor of accreditation visits
8	Professor	Medicine Medical Education New Zealand	Facilitator accreditation visits Active role in accreditation of medical training programme
9	Professor	Medicine Medical Education Iran	Accreditation research Member of national accreditation system Surveyor and facilitator of accreditation visits
10	Professor	Paeds Medicine Medical Education Malaysia	Coordinated accreditation visits Indirect involvement in development of accreditation standards
11	Professor	Emergency Medicine Medical Education PhD Canada	Accreditation research Facilitator of accreditation visits Surveyors’ training
12	Professor	Emergency Medicine Medical Education USA	Facilitator accreditation visits Surveyors’ training
13	Professor	PhD Medical Education General Surgery Pakistan	Facilitator university accreditation visits
14	Associate Professor	Medical Education Caribbean region	Quality assurance and accreditation

For participants who accepted the invitation, a subsequent email was sent containing the study details, including a topic summary, written consent form, and interview guide. The principal investigator conducted one-on-one, semi-structured interviews with experts using Zoom Video conferencing, and the recordings were stored on a password-protected laptop. The interview guide was developed based on the constructs identified in the literature review, such as contextualisation, implicit and explicit mention of equity in accreditation discourse, surveyor training, and accreditation tools. The constructs were operationalised into open-ended questions and subsequently discussed and validated by all members of the research team. Minor refinements were made during the pilot interviews with two potential respondents to improve the questions’ clarity and flow.

### Research Approval:

As part of a doctoral research project, this study was approved by the Advanced Studies & Research Board (ASRB) of Khyber Medical University (7-1/IHPER/PhD-HPE/KMU/23-01; dated: April 5, 2023).

### Data analysis:

The interviews were transcribed verbatim, and inductive and deductive thematic analyses were conducted using the six-step systematic thematic analysis approach proposed by Naeem et al.^25^ This approach expands upon Braun and Clarke’s (2006) methodology, offering enhanced structure and clarity, particularly in the later stages of theme development, conceptualisation through interpretation, and the formulation of conceptual frameworks. As researchers’ synthesis of data may be affected by their preconceptions in the development of themes which could affect the final results,^26^ we used the social constructivist paradigm to analyse the data, considering that reality is subjective and can be interpreted from multiple perspectives.^27^ The personal experiences of the participants were taken as the starting point, and inductive and deductive reasoning were employed^28^ to interpret the meaning of the participants’ responses. The authors thoroughly examined the transcripts to get a feel for the context and content and then narrowed down to key words and phrases.^25^ Our study adhered to the Standards for Reporting Qualitative Research (SRQR) to ensure transparent and rigorous reporting of the study results.^29^ We followed Lincoln and Guba’s criteria to ensure trustworthiness and strengthen the rigor of the study. Credibility was achieved through member checking, and selection of participants from various geographical contexts enhanced transferability. Confirmability was enhanced by cross-referencing the existing literature and incorporating participants’ quotes to ensure that the interpretations remained grounded in the data.

### Theoretical framework to guide the data analysis:

In our study, we adopted an inductive approach to application of theory during the later stages of the data analysis.^30^,^31^ Aristotle’s Theory of Equity served as the theoretical framework for data analysis because of its relevance to recognition of the contextual differences, moral judgment and flexibility in the application of the law (accreditation standards in our research). The central constructs of Aristotle’s theory include general justice, particular justice, the equitable person, and practical wisdom.^13^,^14^ The literature suggests that in exploring healthcare quality improvement, we can gain insights from fields such as education and criminal justice, which have extensive experience in analysing complex social interventions.^32^ Equity is regarded as a multifaceted concept centred on impartiality and tailored support, while “contextualisation” emphasises diverse circumstances.^33^ Initially, we interpreted the data in an open and exploratory manner, utilising a systematic and refined version of Braun and Clarke’s thematic analysis approach,^34^ as theorised by Naeem et al.,^25^ which enabled the identification of the preliminary themes. Subsequently, we conducted a theory-informed analysis, selecting Aristotle’s theory of equity as theoretical framework based on our initial impressions of the data and engagement with the literature.^31^

The initial coding of the first three interviews was conducted individually by all members of the research team. Constructs of the theory of equity were employed to discern how experts conceptualised equity within BME accreditation standards at both the global and national levels. Each team member created an Excel spreadsheet containing codes derived from representative quotations, keywords, or phrases that emerged through identification of recurring patterns and terms in the transcript. Representative quotations were selected based on robust patterns identified in the data, to reflect the diverse viewpoints of participants while maintaining a balance between readability and authenticity.^35^,^36^ Following this preliminary phase, the team convened an online meeting to discuss and reach a consensus on common coding patterns. For example, the code “equity to achieve equality” encapsulates key phrases such as “equal application of standards,” “equality at the school level”, “local adaptation,” and “equity-based recognition criteria.” This code represented a constructive interpretation grounded in the equitable application of law, as conceptualised by Aristotle’s theory of equity. Similarly, the code “recognising honest efforts”, as seen from the lens of the theory of equity, reflects the role of surveyors as “equitable persons.” The themes were meticulously extracted, ensuring that they were reciprocal, recognisable, responsive, and resourceful in relation to the data.^25^

## RESULTS

The experts engaged in intricate discussions about careful use of the term “equity” in implementing medical education accreditation standards, specifically addressing what equity entails, what it does not, and how it might be achieved. The experiences and perspectives of the experts were categorised into five distinct themes, as presented in Table-II.

**Table-II:** Quotations, keywords, key phrases, codes and themes.

***Theme 1: Equity or equality; what should be preferred***		
Quotations	Keywords/ Phrases	Codes
…at the school level they try to make it mainly equality based on what I’ve observed. But if you look at the WFME recognition criteria and how we implement them, when we do the reviews, we try to make it equity-based without ever having thought of it in those terms. U004	equality at school level equity based WFME recognition criteria	Equity to achive equality
The equal application of standards is key within a region, and certainly the contextual development of standards for a region is essential. U0012	equal application of standards is key contextual development	
I use the phrase “Equity in Equality”. We must achieve equality, perhaps at the national level, which is different from the international level. However, national or local standards must include a minimum level of international requirements. M0010	equity in equality	
I would certainly agree that we do not want the LCME (United States) standards to be applied in country X. That would be wrong. However, I would say that standards like the WFME standards that are very clearly stating that they should be locally adapted. Those I would argue should be applied with local adaptation all around the world. U0012	We do not want the LCME standards to be applied in country X Local adaptation	Local adaptation of global standards
***Theme 2: Quality over quantity & contextualisation***		
Quotations	Keywords/ Phrases	Codes
There are standards that are written…, that are not reasonable for any circumstance, like, you know, you must have X number of publications. Those sorts of standards I reject. U0012	not reasonable for any circumstance	Illogical
………, a building that’s so big or you need 10,000 copies of X in your library. Those I would argue are ridiculous standards and these are standards that should be rejected. U0012	10,000 copies of X in your library ridiculous standards	
Setting standards doesn’t do anything, does absolutely nothing. What makes people tick boxes, it makes people pretend. U006	tick boxes make people pretend	
…whenever I see standards and I’ve seen them in India, for example, where they do specify X number of this or X number of that, that’s not very useful. And it ends up constrain and among other things, constraining innovation, because those standards restrict any flexibility or any creativity. U0012	constraining innovation restrict any flexibility or any creativity.	Innovation hindered
…encourage more context-specific standards or context-responsive standards and then perhaps they could draw on international standards. But they don’t have to, sort of rigidly follow international standards. N008	context specific	Contextualisation
To contextualize something from outside your context, you must first understand what will be contextualised and what your context is. I009	Literacy about context	
But the inspection form does not look at the context, and the inspection forms just look at from the point of view of one flat yardstick. P003	one flat yard stick	
the inspection looks at many physical things and is unable to look at the quality of education being imparted. It looks at the number of professors, it looks at the equipment, it looks at the presence of documentation & that’s about it and that’s the limitation that they cannot judge whether the quality training is going on or not. There is a lot of emphasis on quantity, but it is very difficult to gauge quality. P003	lot of emphasis on quantity	Quality or quantity
***Theme 3: Equity is not lowering the standards***		
Quotations	Keywords/ Phrases	Codes
So, I am with equity but for example, we are saying that we are going to accept the lower standard because he is coming from a college with less resources no! I don’t accept that. Students must have the same level of competency. K003	same level of competence	Competence
I would say that equity doesn’t require necessarily relaxing a standard. I think there are things that we can be flexible about but it’s not lowering it. C007	but it’s not lowering it	
We cannot say that a student who has graduated from a rural area must have some relaxation. M0010	cannot have relaxation	
Making different standards for different schools is not justifiable P0013	not justifiable	Unjust
I think they are mainly looking at it from legal perspective, because they don’t want to be taken to court by some other school saying that you had lower standards for this school…. N0014	legal obligation	
***Theme 4: Equity is flexibility in achieving the standards***		
Quotations	Keywords/ Phrases	Codes
…. it might require flexibility in how a standard is demonstrated. So, there’s more than one way to demonstrate that a standard is met is what I would say. And so local context should be taken into account. C007	more than one way	multiple ways to demonstrate a standard
There was one accreditor you know they had standards that could be interpreted in multiple ways. And they said that they did that on purpose because they wanted to give the schools a lot of leverage and how they interpreted them. U004	a lot of leverage to interpret	
We should emphasise and discuss, a continuous dialogue about finding the optimal point between two extremes of contextualization and standardization…. 0019	optimal point	
If the standards are phrased to allow for different ways of getting to the same endpoint, then equity could be achieved through this even the schools are different, and they have so many challenges. U004	different ways of getting to the same endpoint,	
We don’t want all the programs to be perfect as long as you are trying to solve the problem, you will reach (the standard) one day. So, we have accredited programs from colleges or universities in the rural areas because we saw an honest effort to improve the graduates’ outcome. K003	an honest effort	recognizing honest efforts
I mean standards should provide a room to identify the colleges who are at a very basic level compared to those colleges who are operating at the higher level. When you develop these standards like that you are introducing the concept of equity, excellence and at the same time creating positive, healthy competition among colleges. E001	very basic level higher level	Core vs aspirational standards
…but we could have a system by which there would be an agreed upon set of basic standards that everybody would have in the accreditation system and then WFME could just look at them. C0011	set of basic standards	
Identify the core standards and 1st ask your schools to achieve those before going into next. so again, the core should not be determined by the geographical area or the resources. S005	core should not be determined by the geographical area or the resources	
***Theme 5: Best practices to achieve equity***		
Quotations	Keywords/ Phrases	Codes
You should read different versions, statements and documents. For example, you could review the standards for Postgraduate Medical Education, you could review the standards of ACGME of the United States, …. and you should review others. You should review the available local standards that you have. I009	review	Think critically
Try to be analytical. Social science is about argument, analysis, and understanding what you’re doing? and why you’re doing it, and that would be the same with setting up collaborations or anything else. U006	be analytical	
Optimal standards mean the best achievable level of quality, considering your local context. I009	optimal point	
I think we have to have forums for sharing experiences and practices. K003	experiences and practices	Sharing
I think that we have to go back and say OK well which standards are really linked directly to the quality of graduates and which standards are more like you know would be nice to have but they’re not really contributing to a whole lot. C0011	linked directly to the quality of graduates	Link to quality & health system
…. no matter what you develop, standards must be aligned with the kind of health system and the services that you are providing. E001	link them (the standards) with the health system	
…and that’s what accreditation is all about. That is to help the program fulfil its goals and reach its highest potential. K003	help the program	Additional support
We just alter the boxes that they stand on. So, that’s not compromising the standards, but it is altering how much support you get, that would be my favourite model. N008	altering how much support	

### Theme-I: Equity or equality: what should be preferred:

Participants observed that while there is an equality-based implementation of the same set of accreditation standards across regions, the WFME global standards approach is implicitly equity-oriented. The WFME does not impose standards on regions or countries but rather stresses the importance of the thought process in developing contextual standards. An expert with an experience as member of WFME review team noted that:

“…. in a WFME review, the reviewers are looking for in a country is they have the standards, and they put thought into developing them and implementing them in a way that makes sense in that context”. U004

The tension between standardisation and contextual flexibility is reflected in phrase like *“equal application of standards is key within a region.”* One expert regarded the different standards for different schools as unjustifiable because accreditation is meant to enhance the quality of all medical graduates.

“If you start making different standards for different medical schools, that would not be justifiable for the provision of medical knowledge”. P013

An expert introduced the phrase “*equity in equality”*, suggesting that achieving equal application of standards at the national level requires equity-oriented strategies by the accrediting authorities.

### Theme-II: Quality over quantity & contextualisation:

Prescriptive and quantity-focused criteria constrain innovation and hinder creativity, particularly in resource-variable settings. Experts cautioned against the rigid, illogical adaptation of international standards without considering ground realities.

“And I agree that standards that do say you need, you know, a building that’s so big or you need 10,000 copies of X in your library. Those I would argue are ridiculous standards and these are standards that should be rejected” U0013

One expert criticised the current practice of developing local standards as *“one flat yardstick”* to assess regionally diverse institutions, rather than to enhance the quality of educational processes.

“But the inspection form does not look at the context and just look at from the point of view of one flat yardstick, so it is using the principle of equality and not equity”. SM002

Similarly, considering the entire set of foreign accreditation standards and applying them blindly, without regard to the local context, could be counterproductive.

“I think in a good set of quality assurance standards equity and equality are equally important. That is why you cannot really copy and paste standards from the UK, USA, or France and apply them to our countries because the context is different”. S005

### Theme-III: Equity is not lowering the standards:

Experts consistently emphasised that equity in accreditation standards does not entail compromising competence or quality. Instead, equity involves ensuring that all students achieve the same standards of competence irrespective of their institutional or geographical backgrounds. An expert with an extensive experience in developing accreditation standards and as a surveyor at schools with varied contexts observed that:

“We will not relax as far as knowledge, skills, and values are concerned. But the relaxation may be probably about the research quality. I mean I do not expect them (rural medical schools) to publish scientific reports in Nature or in the New England Journal of Medicine, but they need to be community-sensitive to address the issues of their local community.” K003

When the interviewer used a fence analogy to differentiate between equity and equality, suggesting that equity involves providing additional support to those at a disadvantage, one expert responded by noting that the interpretation of equity and equality can vary depending on one’s perspective.

“It depends on how we conceptualize equity in accreditation but if we take the first view that the standards are the same, but some people will need more support than others then I would say that its equity based. If we take the other view, we should lower the standard for some groups that are disadvantaged, but that is not what currently happens. So, if I suppose we take people under that definition that would be equality-based.” N008

However, one expert noted that the accreditation authorities are legally obliged to have uniform set of evaluation criteria for all medical schools. An expert with an experience from Global South accreditation systems, suggested that national accrediting agencies must have a set of core standards, and there should be no compromise on the achievement of those standards, as maintaining a uniform level of expected competence is vital for educational integrity and patient safety.

“For the core standards, the universities should be told clearly that there can’t be any deviation either they have to achieve them, or they have to close the school as they are a threat to patients.” S005

### Theme-IV: Equity is flexibility in interpreting the standards:

Participants underscored the significance of permitting multiple ways to achieve the standards, recognising the diverse institutional contexts and emphasising that “*there’s more than one way”* to achieve the accreditation goal.

“They (the accreditors) wanted to give the schools a lot of leverage in how they interpreted them.” U004

This approach supports equity by enabling institutions with varying resources and challenges to progress towards the same endpoints. Additionally, the participants underscored the significance of “effort and improvement” over perfection, particularly for under-resourced institutions. One expert noted that sometimes the under-resourced school ran an extra mile to cover up their deficiencies, and they could be rewarded by waiving a strict rule or giving time to improve.

“What we can do is sometimes we can waive away something. If they do not have resources for research, for example, if they do not have the budget for research, at least they should show me that they are learning the methods, and once they get the chance, they should do the research. I will not compare a student in Riyadh to a student in Jazan”. K003

### Theme-V: Best practices to achieve equity:

Experts emphasised the need for a critical review of various sets of standards and analytical engagement by accrediting authorities before formulating local, context-responsive accreditation standards. Accrediting authorities must be asked how they cater to institutions in rural areas, highlighting the importance of aligning standards with local realities.

“…. What do they think they are doing to improve the quality of medical education in rural areas? Setting standards doesn’t do anything, does absolutely nothing. What makes people tick boxes, it makes people pretend”. U006

Although international agencies such as the WFME have moved from a prescriptive to a principle-based approach, the division of standards into basic or core and aspirational or excellence standards at the national level may still be required to achieve minimum standards, as indicated by experts from the Global South. This division (at national level) into core and aspirational standards was also suggested by an expert from Global North, considering the accreditation process has become progressively cumbersome.

“The standards are getting higher and higher, and everything is incorporated in those standards like every social issue seems to get its way into the standards and the bar to meet the standard gets higher and higher.” C0012

One of the factors prompting accreditation systems to raise their standards is the pressure by resource-rich schools.

“I find that when you have rich schools or schools that are better resourced, and who constantly push to do more, then what it does? This encourages accreditation systems to raise their own standards. So, they think it’s OK well …now these schools are doing it so let’s do it, which makes it even more difficult for the schools that are less resourced.”C0012

Another recommendation for the accrediting agencies that emerged was to strive for an optimal balance between the pressures of contextualisation and standardisation, while formulating and phrasing accreditation standards. The role of collaborative forums to share experiences during implementation phase could bridge gaps in implementation across diverse contexts.

**Fig.1:**
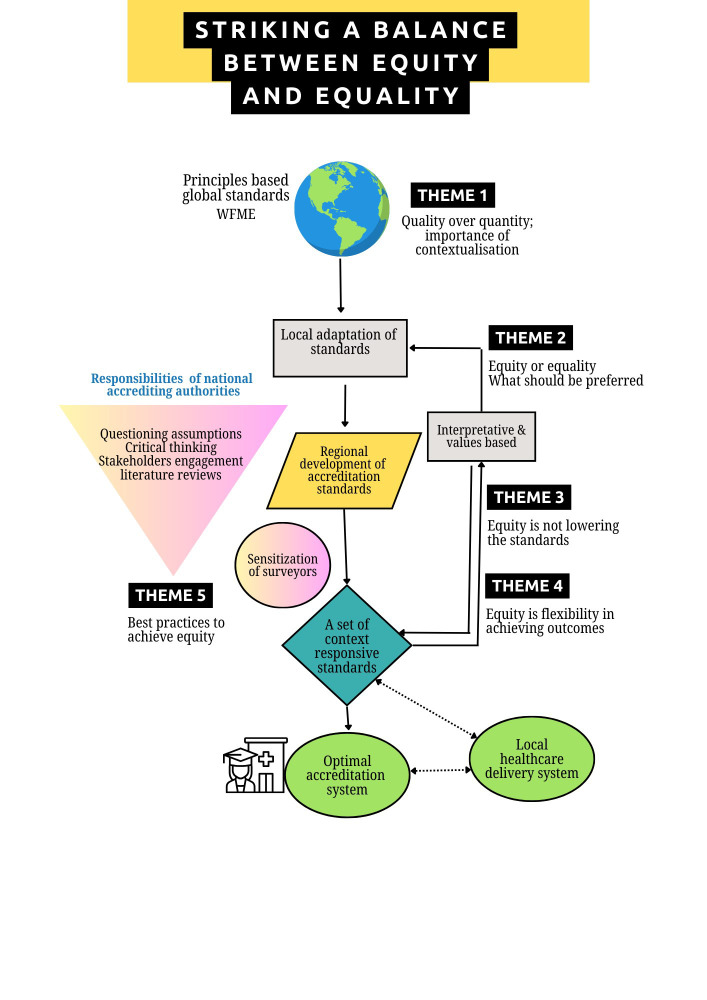
The conceptual model of “equity to achieve equality”.

The themes were synthesised to construct a conceptual model explaining the importance of balancing “equity and equality” to ensure the acceptable quality of medical graduates (Fig.1). This framework demonstrates that transitioning from an international to a national context necessitates equity-based decisions, implying that principles and values are derived from international or global standards. At the national or regional level, accrediting authorities bear the responsibility of critically engaging stakeholders and developing a set of standards that, while not varying across different resource settings, are formulated through an equity lens to ensure their applicability across all contexts. The prevailing practice predominantly involves the formulation of standards that prioritise quantity over quality, particularly in the setting of the Global South.

## DISCUSSION

Our findings coalesced into a conceptual model, yielding insights into the significance of striking a critical balance between equity and equality in the context of BME accreditation standards. While the literature addresses the adaptation of global standards to local contexts,^10^,^37^,^38^ it is essential to acknowledge that contexts can differ markedly, even within the same region or country. Experts had consensus that contextualisation, not standardisation should serve as the foundational principle in the development and implementation of BME accreditation standards at national level. The concept of “equity to achieve equality” emerged as a central theme, emphasising the accrediting authorities to evaluate the medical schools considering contextual factors while achieving the standardised outcomes. Literature endorses this fact that to foster a culture of continuous quality improvement, a standardised yet flexible approach is needed to ensure equitable assessment of medical institutions.^9^,^10^

Experts agreed that the unmodified adaptation of accreditation standards from other contexts is often counterproductive. Consistent with this opinion, a study identified four major categories of differences between international reference standards and national standards, emphasising the variability of local context.^9^ Experts assert that although contextual factors hold significance, the uniform application of standards is essential due to legal obligations, potential biases, and ensuring that all graduates have uniform acquisition of intended outcomes. This is supported by the literature discussing constant tautness between contextualisation and standardisation.^9^,^39^,^40^

While there is substantial literature highlighting the challenges of uniform application of standards across various contexts within the regions,^9^ the role of accrediting bodies in formulating these standards, particularly in considering factors such as resources and the geographical location of medical schools, has been discussed variedly. This reflects a classical Aristotelian equity dilemma: the application of a general rule (standards) in a way that undermines its intended purpose (educational quality). Aristotle recognised that laws are inherently general and cannot foresee every individual case; thus, justice sometimes requires going beyond the letter of the law to achieve its spirit.^12^

The literature indicates that applying a “one-size-fits-all” approach to evaluate medical schools with varying resources^41^ raises concerns about the validity and reliability of the survey instrument.^42^ A strictly numerical instrument poses significant threats to the validity of the survey instrument, a concern also highlighted by the experts in our study. The literature on higher education accreditation endorses this finding by proposing six principles for creating acceptable, professional standards in education: concise, clear, specialised, contextualised, centred on teaching and learning, and supported by robust assessments.^43^

From an Aristotelian perspective, the impracticality of the universal and rigid application of accreditation standards aligns with the idea, that rules intended to promote justice, may become unjust when enforced in a rigid and context-insensitive manner.^14^ Our study suggests that when accrediting agencies establish standards that primarily emphasise quantification and numerical assessment, standards are often perceived as lacking credibility. Numerous studies endorse this finding, indicating that such standards neglect contextual factors and fail to ensure quality; instead, they serve as a source of demotivation for faculty and stakeholders.^44^-^46^ Experts visualized “equity” as a tool that ensures fairness by enabling institutions to achieve comparable outcomes through innovative strategies. Thus, medical education systems can navigate the challenges of globalisation while remaining innovative and responsive to local contexts.^9^

Our most intriguing finding is that achieving equity at the regional level does not mean lowering the standards or creating different set of standards for different contexts. Instead, it calls for accrediting authorities to design standards that can be interpreted and demonstrated in multiple ways to achieve the common goal of ensuring graduate quality. Here, the role of accrediting authorities becomes critical, as the Global South faces a multitude of challenges, in pursuit of achieving international recognition in healthcare education.^5^,^47^-^50^ While accreditation must ensure baseline competence, overly prescriptive processes may stifle innovation, particularly in under resourced settings. This aligns with critiques of “accreditation fatigue” where bureaucratic demands overshadow educational outcomes^10^,^51^ and issue of evaluability that regards standards as consensual statements and principles rather than numerical indicators.^18^

Our findings also highlight progress-based concept of accreditation, stressing upon recognising “honest efforts” of the institutions to improve graduate outcomes. Experts advised that surveyors might recommend waiving certain requirements when they observe genuine efforts from the school. This aligns with the Aristotle’s theory of equity,^14^ which advocates balancing rigor and fairness by acknowledging the efforts of programs that may not meet every standard due to constraints but still deliver quality education. However, such waivers are contingent upon extensive and detailed data triangulation during survey visits, which must demonstrate the institution’s genuine efforts. It also emphasises the role of surveyors as equitable persons *(epiekis)*,^52^ decision-makers, or someone who can discern when and how to apply equity.

Therefore, comprehensive training and assessment of surveyors are essential to ensure competence and process uniformity.^18^,^53^ This is particularly true when the accrediting instrument is unable to discern the implicit reasoning of the surveyor while evaluating the school against a specific checklist as they are not required to justify their judgments.^42^ Aristotle’s theory of equity explains this by the concept of *“epieikeia”* as a virtue to bridge the gap when it falls short by allowing judges (surveyors) to consider relevant mitigating factors and determine what a just decision requires beyond the strict application of the law.^12^ Our findings further extend this argument by advocating for core standards at the national level for all schools, irrespective of geographical location and resources, while also suggesting aspirational benchmarks.

The alignment of accreditation standards with local healthcare delivery services is important for effective and contextual implementation of accreditation standards. This finding is supported by Ahmed and Vellani,^54^ who proposed strategies to address challenges like private medical schools’ proliferation, declining standards, and the migration of graduates. The study proposed enhancing district health services, incorporating medical education practical experiences within these services, and implementing national accreditation standards that link education and service quality.^54^

Our findings also indicate that defining optimal standards, is a logical way to balance the constant tension between equity and equality in the practical implementation of accreditation standards. Optimal standards acknowledge the tensions between the two extremes of standardisation and contextualisation. Defining optimal standards according to literature is a challenging task, especially in the heterogeneous contexts,^37^,^55^ due to conflicting interests of various stakeholders’ groups.^56^ For instance, scholars and academics often set higher standards, while administrators and institutions tend to advocate lower standards. In line with this, Taber et al. defined a framework for medical education accreditation that emphasised key design decisions to ensure alignment of accreditation systems with local context, indicating that universal set of best practices does not exist.^18^

To ensure that accreditation systems remain adaptable to the swiftly evolving demands of healthcare education and sociocultural transformations, it is imperative to guarantee flexibility, and continuous education about the process. For example, in Malaysia, the Ministry of Higher Education (MoHE) conducts workshops to educate institutions on self-evaluation.^57^ Other examples include distributing pamphlets on accreditation requirements at symposia, discussions, and educational conferences as well as making them accessible through websites.

### Strengths and limitations:

The study draws on qualitative in-depth interviews with the accreditation experts from diverse geographical and regulatory contexts including Pakistan, United States, Canada, New Zealand and Saudi Arabia. This enabled the exploration of diverse perspectives from both Global North and Global South settings that enhance the conceptual breadth of the analysis. However, despite efforts to recruit participants from a wider range of low resource settings experts from Sudan and some part of Africa could not respond to our request resulting in possible underrepresentation of certain Global South perspective. This may have limited the extent to which the findings capture the full diversity of challenges faced across all LMIC context. In addition, relatively small sample size while consistent with qualitative inquiry constrains the transferability of findings. The study design also relies on interpretive judgments rather than direct observation of accreditation practices which may be influenced by participants professional roles and contextual positioning.

## CONCLUSION

Developing different set of standards for different schools is unjustifiable, challenging and contrary to the principle of fairness. However, the concept of “equity to achieve equality” through differentiated support reaffirms that equity aligns with promoting high standards in education. By providing context-specific scaffolding, embracing flexibility (equity to achieve equality), systematic surveyor training, and collaborative improvement, accrediting agencies can foster inclusive excellence across diverse medical education landscapes.

## REFLEXIVITY

NS is a PhD (HPE) Scholar and serves in the Department of HPE at a private undergraduate medical school. Two members (UM and AS) had doctorates in HPE and experience in accreditation visits and the formulation of accreditation standards. MI holds a doctorate in mental health and an additional CHPE qualification. As a member of the institutional team, MI has experience in conducting accreditation visits and formulating institutional policies. The research team’s experience in conducting accreditation visits may lead us to interpret findings through the lens of practices in Low Middle-Income countries (LMIC), potentially overlooking issues of well-off countries.

To address these possible influences, we implemented a systematic and transparent approach according to a standard protocol for the selection, screening, synthesis, and analysis of the literature. Additionally, to critically assess our biases and ensure that our conclusions were thorough and impartial, we held frequent reflexive discussions.

### Authors’ contribution:

**NS:** Conceptualized the study as a part of her doctoral research project.

**UM, AS and MI:** refined the conceptual depth and provided the intellectual oversight.

**UM, AS and MI:** Provided critical feedback and approved the study.

**NS:** Did the data collection and transcription. All authors engaged in coding and data analysis.

**NS and MI**: Wrote the draft manuscript.

**UM and AS:** Critically reviewed the draft and made corrections.

All the authors were involved in writing, editing and approving the final version of the manuscript.

All authors are responsible for integrity and accuracy of the study findings.
